# Oxidative Stress and Gut-Derived Lipopolysaccharides in Neurodegenerative Disease: Role of NOX2

**DOI:** 10.1155/2020/8630275

**Published:** 2020-01-31

**Authors:** Lorenzo Loffredo, Evaristo Ettorre, Anna Maria Zicari, Maurizio Inghilleri, Cristina Nocella, Ludovica Perri, Alberto Spalice, Chiara Fossati, Maria Caterina De Lucia, Fabio Pigozzi, Mauro Cacciafesta, Francesco Violi, Roberto Carnevale, Neurodegenerative Disease study group

**Affiliations:** ^1^Department of Internal Medicine and Medical Specialties, Sapienza University of Rome, Italy; ^2^Department of Cardiovascular, Respiratory, Nephrologic, Anesthesiologic and Geriatric Sciences, Division of Gerontology, Sapienza University of Rome, Rome, Italy; ^3^Department of Pediatrics, Sapienza University of Rome, Italy; ^4^Department of Human Neuroscience, Sapienza University, Rome, Italy; ^5^Department of Medical-Surgical Sciences and Biotechnologies, Sapienza University of Rome, Latina, Italy; ^6^Cardiocentro Mediterranea, Napoli, Italy; ^7^Department of Movement, Human and Health Sciences, University of Rome “Foro Italico”, Italy; ^8^UTNA, IRCCS Neuromed Pozzilli, Isernia, Italy

## Abstract

**Background:**

Neurodegenerative diseases (ND) as Alzheimer's disease, Parkinson's disease, and amyotrophic lateral sclerosis represent a growing cause of disability in the developed countries. The underlying physiopathology is still unclear. Several lines of evidence suggest a role for oxidative stress and NADPH oxidase 2 (NOX2) in the neuropathological pathways that lead to ND. Furthermore, recent studies hypothesized a role for gut microbiota in the neuroinflammation; in particular, lipopolysaccharide (LPS) derived from Gram-negative bacteria in the gut is believed to play a role in causing ND by increase of oxidative stress and inflammation. The aim of this study was to assess NOX2 activity as well as serum 8-iso-prostaglandin F2*α* (8-iso-PGF2*α* (8-iso-PGF2

**Methods:**

One hundred and twenty-eight consecutive subjects, including 64 ND patients and 64 controls (CT) matched for age and gender, were recruited. A cross-sectional study was performed to compare serum activity of soluble NOX2-dp (sNOX2-dp), blood levels of isoprostanes, serum H_2_O_2_, and LPS in these two groups. Serum zonulin was used to assess gut permeability.

**Results:**

Compared with CT, ND patients had higher values of sNOX2-dp, 8-iso-PGF2*α* (8-iso-PGF2*p* < 0.001), zonulin (Rs = 0.411; *p* < 0.001), zonulin (Rs = 0.411; *p* < 0.001), zonulin (Rs = 0.411; *α* (8-iso-PGF2*p* < 0.001), zonulin (Rs = 0.411; *p* < 0.001), zonulin (Rs = 0.411; *α* (8-iso-PGF2*p* < 0.001), zonulin (Rs = 0.411; *β*, 0.459; *p* < 0.001), zonulin (Rs = 0.411; *α* (8-iso-PGF2*β*, 0.459; *p* < 0.001), zonulin (Rs = 0.411; *R*^2^ = 57%).

**Conclusion:**

This study provides the first report attesting that patients with ND have high NOX2 activation that could be potentially implicated in the process of neuroinflammation.

## 1. Introduction

Neurodegenerative diseases (ND) as Alzheimer's disease (AD), Parkinson's disease (PD), and amyotrophic lateral sclerosis (ALS) represent an increasingly frequent cause of disability in the developed countries [1]. Growing evidences demonstrated that oxidative stress plays a pivotal role in the initiation and progression of ND [2]. In particular, there is emerging experimental evidence that reactive oxygen species (ROS), derived from NADPH oxidase 2 (NOX2), are important in apoptotic pathways and in mediating the inflammatory responses in the central nervous system [3, 4]. NOX-dependent ROS production can be detected in microglia, astrocytes, neurons, platelets, and endothelial cells [3, 4]. Several animal studies showed increased NOX2 activation associated with O_2_^−^ production and cognitive decline; furthermore, a close relationship between NOX2 activity and the levels of *β*-amyloid and with degeneration of dopaminergic neurons was found [3, 4]. Platelet, endothelial cells, and lymphocytes could also contribute to the deterioration of the central nervous system [3, 4]. Activated platelets are implicated in the neurodegenerative process by the production of amyloid precursor protein (APP) and beta-amyloid peptide (A*β*) by thrombosis of large and small vessels in the cerebral arterial district [5]. NOX2 activation is associated with increased platelet aggregation along with endothelial dysfunction [6–9]. Furthermore, the oxidative stress generated by NOX2 activation promotes activation, proliferation, and differentiation of T cells (as increase in Th1 and Th17 response) that could contribute to the inflammation and to the neurodegenerative process [10].

Nevertheless, the physiologic and pathophysiologic roles of such NOX enzymes in ND are only partially understood, and to the best of our knowledge, there is no study that assessed the activation of NOX2 in patients with AD, PD, and ALS. We speculated that ND subjects have NOX2 overactivation and increased oxidative stress that may contribute to neurodegenerative process; thus, in this study, we wanted to evaluate NOX2 activation and 8-iso-PGF2*α* production in serum of ND patients and controls.

In human, the gut microbiota plays pivotal functions as intestinal epithelial barrier protection, immune homeostasis, immune responses (as induction of T cell-dependent and independent production of IgA antibodies, promotion of mucosal Th17 cell response and IL-10 from intestinal macrophages), and protection against pathogen colonization [11].

To better understand a potential cause of NOX2 activation, we analyzed also the gut microbiota in this population. Recent studies suggested that changes of gut microbiota are associated to neuroinflammation [12]. In particular, lipopolysaccharide (LPS) derived from Gram-negative bacteria is believed to play a role in causing ND by increase of oxidative stress and inflammation [12, 13]. A relationship between LPS and NOX2 activation, in other clinical settings such as NAFLD [14], pneumonia [15], and atherosclerotic plaque [16], has been previously described. Thus, we assessed the association between Nox2 and LPS serum levels to evaluate a potential role for gut-derived LPS in eliciting systemic Nox2 activation. Furthermore, to assess the relationship between NOX2 activation and systemic oxidative stress, we evaluated the serum 8-iso-prostaglandin F2*α* (8-iso-PGF2*α*), stable end products of lipid peroxidation of arachidonic acid, that have been found elevated in a number of conditions such as cardiovascular and neurodegenerative diseases [17] and serum H_2_O_2_.

## 2. Materials and Methods

One hundred and twenty-eight consecutive subjects, who were referred to the Neurologic Department, to the Geriatric Clinic and at the Department of Internal Medicine, and to the Medical Specialties of “Sapienza” University of Rome from January 2018 to November 2018, were enrolled in this study. Forty-seven subjects were affected by AD (20 males and 27 females, mean age 75 ± 8), 9 by ALS (4 males and 5 females, mean age 66 ± 5), and 8 by PD (6 males and 2 females, mean age 70 ± 5).

Sixty-four control subjects (36 males and 28 females, mean age 72 ± 8), matched for aged and gender, were enrolled at the Department of Internal Medicine and Medical Specialties at Sapienza University.

Active smoking was defined if the subjects had smoked any cigarette in the last 3 months.

The following exclusion criteria for all subjects are included: existence of renal disease, malignancy, treatment with immunosuppressive drugs or antioxidants, liver failure, and acute disease.

Informed consent was obtained from all subjects; the study conformed to the ethical guidelines of the 1975 Declaration of Helsinki and was approved by the Sapienza University of Rome Ethics Committee.

### 2.1. Blood Sampling

Blood sampling was collected between 8:00 and 9:00 am for routine biochemical evaluations, including fasting total cholesterol and glucose, and for oxidative stress analysis. Blood samples were collected in Vacutainers (Vacutainer Systems, Belliver Industrial Estate, Plymouth, UK) after an overnight fast (12 hours). Samples were centrifuged at 300g for 10 minutes, and the supernatant was collected and stored at -80°C until dosage. Cholesterol analysis was assessed by an enzymatic colorimetric method on a Dimension RXL apparatus (Dade Behring AG, Ziegelbrücke, Switzerland).

### 2.2. ELISA Detection of sNox2-dp

NOX2-derived peptide, a marker of NADPH oxidase activation, was assessed in serum by ELISA method. The peptide was recognized by the specific monoclonal antibody against the amino acidic sequence (224–268) of the extra membrane portion of Nox2 (catalytic core of NADPH oxidase). Intra-assay and interassay coefficients of variation were 5.2% and 6%, respectively.

### 2.3. Serum H_2_O_2_

The production of H_2_O_2_ was evaluated by a colorimetric assay (Arbor Assay, Ann Arbor, Michigan) and expressed in *μ*M. Intra-assay and interassay coefficients of variation were 2.1% and 3.7%, respectively.

### 2.4. Serum 8-iso-Prostaglandin F2*α* (8-iso-PGF2*α*)

8-iso-PGF2*α* levels were measured in serum by using a colorimetric assay kit (Abcam and DRG International, Inc.).

### 2.5. Serum Zonulin

Serum zonulin levels were measured using a commercially ELISA kit (Elabscience). Antibody specific for zonulin has been precoated onto a microplate and 100 *μ*l of standards, and patient serum samples were added and incubated 90 min at 37°C. Then, a biotinylated detection antibody specific for zonulin and Avidin-Horseradish Peroxidase (HRP) conjugate was added to each microplate. The amount of zonulin was measured with a microplate autoreader at 450 nm. Values were expressed as ng/ml; both intra-assay and interassay coefficients of variation were within 10%.

### 2.6. LPS

Plasma samples were thawed only once and used to perform specific sandwich enzyme-linked immunosorbent assay (ELISA) to measure LPS (Hycult Biotechnology, Uden, Netherlands). The kit has a concentration range of 0.04 to 10.0 EU/ml.

#### 2.6.1. LPS Detection by LAL Chromogenic Endotoxin Quantitation Kit

Gram-negative bacterial endotoxins were detected by Pierce LAL Chromogenic Endotoxin Quantitation Kit (Thermo Fisher Scientific). Briefly, bacterial endotoxin catalyzes the activation of a proenzyme in the modified Limulus Amebocyte Lysate (LAL). The activated proenzyme then catalyzes the splitting of *p*-Nitroaniline (pNA) from the colorless substrate, Ac-Ile-Glu-Ala-Arg-pNA; the activation rate is proportional to the sample endotoxin concentration. After stopping the reaction, the released pNA is photometrically measured at 405-410 nm. The correlation between absorbance and endotoxin concentration is linear in the 0.1-1.0 EU/ml range.

### 2.7. ADL and IADL

The scales for investigating functional autonomy Activities of Daily Living (ADL) and Instrumental Activities of Daily Living (IADL) were performed as previously described [18]. ADL is a 6-item scale [19] that investigates the independence in basic actions of daily living like personal hygiene, dressing, toileting/continence, transferring, and eating. IADL is an 8-item scale [20] that investigates autonomy in complex activities for functioning in community settings like using the telephone, shopping, caring of the house, travelling in the city, managing drugs, and finances.

### 2.8. Statistical Analysis

Statistical analyses were undertaken using SPSS 18.0 software for Windows (SPSS, Chicago, IL, USA). The Kolmogorov-Smirnov test was used to determine whether variables were normally distributed. Normally distributed data are described as means ± standard deviations (SDs). Between-group differences were analyzed by Kruskal-Wallis tests (for nonnormally distributed data) or analysis of variance (ANOVA). Differences between percentages were assessed by the *χ*^2^ test. Bivariate analysis was performed by Spearman's correlation; the variables with evidence of an association *p* < 0.10 were included in a multivariable linear regression using an automated procedure with forward selection. A *p* value of <0.05 was considered statistically significant.

### 2.9. Sample Size Determination

In this cross-sectional study, sample size calculation was computed with respect to a two-tailed Student's *t*-test for independent groups, considering 11 pg/ml (*δ*) as difference for Nox2 levels between neurodegenerative patients and controls, 20 pg/ml as S.D., 0.05 (*α*) as type I error probability, and 0.90 as power 1-*β*. The minimum sample size was *n* = 50 patients/group.

## 3. Results

Clinical characteristics of patients with ND and controls are reported in the table. No significant difference between the 2 groups was found for age, fasting blood glucose, systolic and diastolic blood pressure, BMI, or smoking (Table 1).

Compared with controls, sNOX2-dp, serum H_2_O_2_, serum 8-iso-PGF2*α*, LPS evaluated by ELISA kit and chromogenic assay, and zonulin were higher in ND (Table 1).

Simple linear regression analysis showed that sNOX2dp was significantly correlated with serum H_2_O_2_ (Rs = 0.329; *p* < 0.001), serum LPS (pg/ml) (Rs = 0.441; *p* < 0.001), serum LPS (EU/ml) (Rs = 0.271; *p* < 0.001), zonulin (Rs = 0.411; *p* < 0.001), and 8-iso-PGF2*α* (Rs = 0.244; *p* = 0.006). Furthermore, LPS significantly correlated with serum zonulin (Rs = 0.818; *p* < 0.001) and 8-iso-PGF2*α* (Rs = 0.280; *p* = 0.001).

Multiple linear regression analyses, including the variables linearly associated with the dependent variable, were performed to define the independent predictors of sNOX2-dp in the overall population. LPS (SE, 0.165; standardized coefficient *β*, 0.459; *p* < 0.001) and 8-iso-PGF2*α* (SE, 0.018; standardized coefficient *β*, 0.220; *p* = 0.005) emerged as the only independent predictive variables associated with sNOX2-dp (*R*^2^ = 57%).

**Figure 1:**
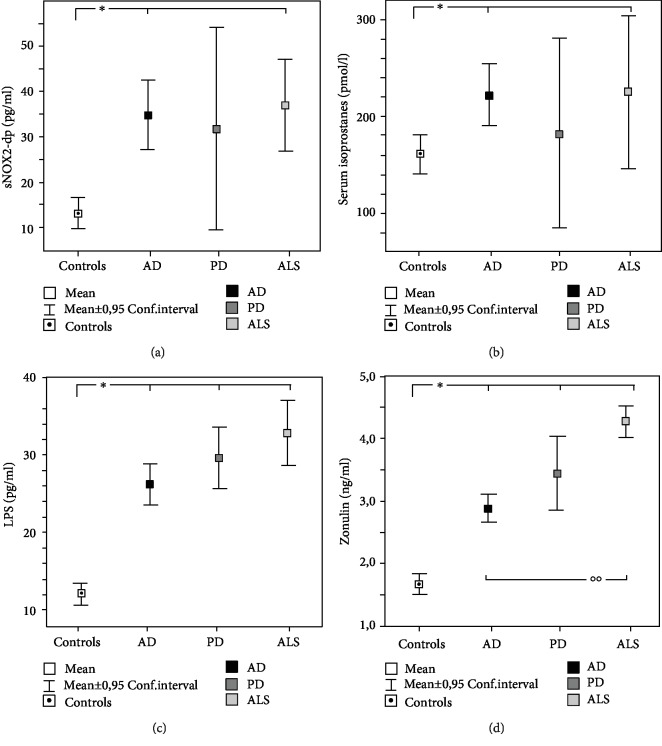
NOX2 (a), serum isoprostanes (b), LPS (c), and zonulin (d) in AD, PD, and ALS patients.

A further analysis among ND patients was performed. Clinical characteristics of AD, PD, and ALS are reported in Table 2. The subgroup analysis, performed with Kruskall-Wallis test, showed significant differences among the groups (Figures 1(a)–1(d)). In particular, compared to controls, sNOX2-dp and isoprostanes were higher in AD and ALS (Figures 1(a) and 1(b)); LPS and zonulin were significantly higher in all the ND groups (Figures 1(c) and 1(d)).

## 4. Discussion

This study provides evidences that patients with ND have high NOX2 activation and suggests a potential role for gut microbiota as source of oxidative stress in this population.

NOX2 is considered not only a key target in atherosclerosis [6, 21, 22] but also a pivotal mediator of the oxidative and inflammatory responses in these neurodegenerative diseases [3]. Reactive oxygen species (ROS) derived from NOX2 activation leads to neuronal oxidative damage (e.g., microglia activation and/or leukocyte infiltration of the central nervous system), favoring the initiation and progression of ND [3]. This is the first study that analyzed the systemic activation of NOX2 in human with ND; to the best of our knowledge, NADPH oxidase activation was studied only in animals [23] and in postmortem studies [24]. Animal model showed that NOX2 is upregulated in several ND as AD, PD, ASL, Huntington's disease, and multiple sclerosis and that apocynin, which is a NOX inhibitor, improved survival and symptoms via decreased neuroinflammation [23].

A postmortem study in AD patients showed that cerebral NOX is upregulated [24]. This study supports and extends this report showing that NOX2 is overactivated in living patients with ND.

Further evidence of the coexistence of oxidative stress in ND patients was provided by enhanced serum level isoprostanes that are stable end products of lipid peroxidation derived from arachidonic acid by a cyclooxygenase-independent mechanism, resulting from the attack of ROS on phospholipids in the cell membranes. Isoprostanes are considered a reliable marker of oxidative stress stemming prevalently from Nox2 activation [6]. This finding is in accordance with a previous study that found increased levels of isoprostanes in ND [25]. Moreover, we found increased H_2_O_2_ production corroborating the role of the increased ROS production in the pathogenesis of chronic ND [26].

Previous studies showed that alteration of gut microbiota could affect NOX2 activation and redox signalling in several animal models [27–29]. Thus, an intriguing hypothesis of NOX2 activation in ND could derive from changes of gut microbiota in ND. Several studied identified dysbiosis within patients suffering from AD, PD, and ALS and proposed the concept of “gut-brain-axis” to explain some ND [30–32]. Gram-negative bacteria of gastrointestinal tract (as Bacteroides fragilis and Escherichia coli) release LPS that exerts proinflammatory actions on neurons [13]. Animal studies showed that systemic LPS administration increases neuroinflammation and leads to progressive neurodegeneration [13, 33]. The mechanism through which LPS damages the brain is unclear; LPS could act on leukocyte and microglial TLR4-CD14/TLR2 receptors to produce NF*κ*B-mediated increases of TNF-*α* and IL-1*β* [34]. However, further studies are necessary to understand this issue.

In accordance with the literature, we found high levels of circulating LPS in patients with ND [34, 35] with a significant correlation between LPS and Nox2. A recent study identified micro-RNA binding sites related to gut bacteroidetes and proteobacteria that could explain the mechanism of lipopolysaccharide biosynthesis in AD and PD [36]; however, the mechanism of LPS increase deserves further investigations.

LPS was found in the central nervous system also; previous studies in brains of AD patients showed that LPS is localized in amyloid plaques and around vessels, suggesting possible sites of direct damage in neurodegeneration [34, 35].

To address if gut permeability may account for LPS increase in ND, we measured the circulating levels of zonulin, which modulates gut permeability by disassembling the intercellular tight junctions [37]. Experimental and clinical studies demonstrated that zonulin upregulation increases gut permeability [38]. The increased serum levels of zonulin in ND patients and its correlation with serum LPS provide the evidence that gut permeability is enhanced in this large spectrum of disease and may be responsible for the high circulating levels of LPS.

Inhibition of NADPH oxidase (by apocynin or deletion for gp91phox or p47) after LPS administration leads to lower neuroinflammation in animals [39]. A NOX2 inhibition (e.g., with antioxidant treatment) might be useful to modulate neuroinflammation in human, but prospective and interventional studies are necessary to establish a cause-effect relationship between LPS- and NOX2-related oxidative stress and ND.

In this study, we showed that Nox2 activation correlates with LPS and isoprostanes; however, further studies are needed to establish the pathophysiological mechanisms responsible for neurodegeneration processes.

The study had some limitations and implications. NOX2 activation and oxidative stress were studied in systemic circulation and not in the brain by biopsies. However, the latter is an invasive and unethical method. Furthermore, we did not evaluate other NADPH isoforms, such as NOX1 and NOX4, that could also contribute to increase oxidative stress.

The mechanism accounting for LPS translocation from gut microbiota to central nervous system was not addressed by the present study; however, changes in gut permeability might be a plausible mechanism as increased serum zonulin, which reflects enhanced gut permeability, significantly correlated with blood LPS. Nevertheless, we cannot exclude that serum LPS could derive also from other sources; thereby, further study is necessary to support this issue.

Future studies will have to analyze the relationship between NOX2 levels and the severity of neurodegenerative disease and the effect of diet, probiotics, or antioxidants on NOX2 activity and LPS in this population.

In conclusion, this study provides the first report attesting that patients with ND have high NOX2 activation that could be potentially implicated in the pathological pathways of neuroinflammation. These findings could be very important premises for both determining preclinical markers of neurodegeneration and developing disease-modifying therapies.

## Figures and Tables

**Table 1:** Clinical and laboratory characteristics of ND and CT subjects.

Clinical characteristics	ND (*n* = 64)	CT (*n* = 64)	*p*
Age (years)	73 ± 6	72 ± 8	0.320
Gender (males/females)	36/28	36/28	1.0
Total cholesterol (mg/dl)	176 ± 55	170 ± 60	0.122
Glycaemia (mg/dl)	92 ± 9	94 ± 11	0.470
Systolic blood pressure (mmHg)	120 ± 12	125 ± 10	0.579
Diastolic blood pressure (mmHg)	75 ± 10	73 ± 12	0.720
BMI	25.0 ± 3.1	25.3 ± 3.2	0.54
Hypertension	46/24	48/16	0.843
Diabetes	7/57	8/56	0.783
Dyslipidemia	28/36	25/39	0.590
Current smoking	2/61	3/61	0.648
Serum isoprostanes (pmol/l)	221 ± 108	152 ± 68	**0.005**
sNOX2-dp (pg/ml)	34 ± 24	13 ± 12	**<0.001**
H_2_O_2_ (*μ*M)	31 ± 8	9 ± 3	**<0.001**
LPS (pg/ml)	27 ± 8	12 ± 6	**<0.001**
LPS (EU/ml)	0.300 ± 0.08	0.149 ± 0.05	**<0.001**
Zonulin (ng/ml)	3.1 ± 0.8	1.6 ± 0.6	**<0.001**
ADL	4.1 ± 1.7	5.9 ± 0.3	**<0.001**
IADL	2.0 ± 1.9	6.4 ± 1.4	**<0.001**

**Table 2:** Clinical and laboratory characteristics of AD, PD, and ALS patients.

Clinical characteristics	AD (*n* = 47)	PD (*n* = 8)	ALS (*n* = 9)
Age (years)	75 ± 8	71 ± 6	67 ± 5
Gender (males/females)	24/23	2/6	4/5
BMI	25.7 ± 3.3	26.4 ± 3.8	24.6 ± 3.5
Hypertension	35	4	7
Diabetes	5	0	2
Dyslipidemia	22	2	4
Current smoking	0	1	0
Serum isoprostanes (pmol/l)	221 ± 110	182 ± 117	253 ± 86
sNOX2-dp (pg/ml)	35 ± 26	32 ± 26	37 ± 13
H_2_O_2_ (*μ*M)	31 ± 9	32 ± 12	31 ± 6
LPS (pg/ml)	26 ± 9	29 ± 5	33 ± 5
LPS (EU/ml)	0.291 ± 0.09	0.310 ± 0.05	0.340 ± 0.06
Zonulin (ng/ml)	2.9 ± 0.7	3.4 ± 0.7	4.3 ± 0.7
ADL	4.4 ± 1.4	—	—
IADL	2.0 ± 1.8	—	—

## Data Availability

The data used to support the findings of this study are available from the corresponding author upon request.

## Abbreviations

NADPH oxidase: Nicotinamide-adenine dinucleotide phosphate oxidase
8-iso-PGF2*α*: 8-iso-Prostaglandin F2*α*
AD: Alzheimer's disease
PD: Parkinson's disease
ALS: Amyotrophic lateral sclerosis
LPS: Lipopolysaccharide.

## References

[1] Fereshtehnejad S. M., Vosoughi K., Heydarpour P. (2019). Burden of Neurodegenerative Diseases in the Eastern Mediterranean Region, 1990-2016: Findings from the Global Burden of Disease Study 2016. *European Journal of Neurology*.

[2] Niedzielska E., Smaga I., Gawlik M. (2016). Oxidative stress in neurodegenerative diseases. *Molecular Neurobiology*.

[3] Sorce S., Krause K. H. (2009). NOX enzymes in the central nervous system: from signaling to disease. *Antioxidants & Redox Signaling*.

[4] Cahill-Smith S., Li J. M. (2014). Oxidative stress, redox signalling and endothelial dysfunction in ageing-related neurodegenerative diseases: a role of NADPH oxidase 2. *British Journal of Clinical Pharmacology*.

[5] Violi F., Loffredo L., Carnevale R., Pignatelli P., Pastori D. (2017). Atherothrombosis and oxidative stress: mechanisms and management in elderly. *Antioxidants & Redox Signaling*.

[6] Violi F., Carnevale R., Loffredo L., Pignatelli P., Gallin J. I. (2017). NADPH oxidase-2 and Atherothrombosis. *Arteriosclerosis, Thrombosis, and Vascular Biology*.

[7] Violi F., Sanguigni V., Loffredo L. (2006). Nox2 is determinant for ischemia-induced oxidative stress and arterial vasodilatation: a pilot study in patients with hereditary Nox2 deficiency. *Arteriosclerosis, Thrombosis, and Vascular Biology*.

[8] Loffredo L., Carnevale R., Sanguigni V. (2013). Does NADPH oxidase deficiency cause artery dilatation in humans?. *Antioxidants & Redox Signaling*.

[9] Violi F., Sanguigni V., Carnevale R. (2009). Hereditary deficiency of gp91^phox^ is associated with enhanced arterial dilatation: results of a multicenter study. *Circulation*.

[10] Solleiro-Villavicencio H., Rivas-Arancibia S. (2018). Effect of chronic oxidative stress on neuroinflammatory response mediated by CD4^+^T cells in neurodegenerative diseases. *Frontiers in Cellular Neuroscience*.

[11] Pickard J. M., Zeng M. Y., Caruso R., Núñez G. (2017). Gut microbiota: role in pathogen colonization, immune responses, and inflammatory disease. *Immunological Reviews*.

[12] Quigley E. M. M. (2017). Microbiota-brain-gut axis and neurodegenerative diseases. *Current Neurology and Neuroscience Reports*.

[13] Zhao Y., Jaber V., Lukiw W. J. (2017). Secretory products of the human GI tract microbiome and their potential impact on Alzheimer’s disease (AD): detection of lipopolysaccharide (LPS) in AD hippocampus. *Frontiers in Cellular and Infection Microbiology*.

[14] Loffredo L., Zicari A. M., Perri L. (2019). Does Nox2 overactivate in children with nonalcoholic fatty liver disease?. *Antioxidants & Redox Signaling*.

[15] Cangemi R., Pignatelli P., Carnevale R. (2016). Low-grade endotoxemia, gut permeability and platelet activation in community-acquired pneumonia. *The Journal of Infection*.

[16] Carnevale R., Nocella C., Petrozza V. (2018). Localization of lipopolysaccharide from Escherichia coli into human atherosclerotic plaque. *Scientific Reports*.

[17] Montuschi P., Barnes P. J., Roberts L. J. (2004). Isoprostanes: markers and mediators of oxidative stress. *The FASEB Journal*.

[18] McKhann G. M., Knopman D. S., Chertkow H. (2011). The diagnosis of dementia due to Alzheimer's disease: Recommendations from the National Institute on Aging-Alzheimer's Association workgroups on diagnostic guidelines for Alzheimer's disease. *Alzheimers Dement*.

[19] Katz S., Ford A. B., Moskowitz R. W., Jackson B. A., Jaffe M. W. (1963). Studies of illness in the Aged. *JAMA*.

[20] Lawton M. P., Brody E. M. (1969). Assessment of older people: self-maintaining and instrumental activities of daily living. *Gerontologist*.

[21] Loffredo L., Zicari A. M., Occasi F. (2015). Endothelial dysfunction and oxidative stress in children with sleep disordered breathing: role of NADPH oxidase. *Atherosclerosis*.

[22] Loffredo L., Zicari A. M., Occasi F. (2018). Role of NADPH oxidase-2 and oxidative stress in children exposed to passive smoking. *Thorax*.

[23] Sorce S., Stocker R., Seredenina T. (2017). NADPH oxidases as drug targets and biomarkers in neurodegenerative diseases: what is the evidence?. *Free Radical Biology & Medicine*.

[24] Ansari M. A., Scheff S. W. (2011). NADPH-oxidase activation and cognition in Alzheimer disease progression. *Free Radical Biology & Medicine*.

[25] Miller E., Morel A., Saso L., Saluk J. (2014). Isoprostanes and neuroprostanes as biomarkers of oxidative stress in neurodegenerative diseases. *Oxidative Medicine and Cellular Longevity*.

[26] Kilbride S. M., Telford J. E., Davey G. P. (2008). Age-related changes in H_2_O_2_ production and bioenergetics in rat brain synaptosomes. *Biochimica et Biophysica Acta (BBA) - Bioenergetics*.

[27] Neish A. S. (2013). Redox signaling mediated by the gut microbiota. *Free Radical Research*.

[28] Alam A., Leoni G., Quiros M. (2016). The microenvironment of injured murine gut elicits a local pro-restitutive microbiota. *Nature Microbiology*.

[29] Karbach S. H., Schönfelder T., Brandão I. (2016). Gut microbiota promote angiotensin II-induced arterial hypertension and vascular dysfunction. *Journal of the American Heart Association*.

[30] Ticinesi A., Tana C., Nouvenne A., Prati B., Lauretani F., Meschi T. (2018). Gut microbiota, cognitive frailty and dementia in older individuals: a systematic review. *Clinical Interventions in Aging*.

[31] Kowalski K., Mulak A. (2019). Brain-gut-microbiota axis in Alzheimer’s disease. *Journal of Neurogastroenterology and Motility*.

[32] Sasmita A. O. (2019). Modification of the gut microbiome to combat neurodegeneration. *Reviews in the Neurosciences*.

[33] Qin L., Wu X., Block M. L. (2007). Systemic LPS causes chronic neuroinflammation and progressive neurodegeneration. *Glia*.

[34] Zhan X., Stamova B., Sharp F. R. (2018). Lipopolysaccharide associates with amyloid plaques, neurons and oligodendrocytes in Alzheimer’s disease brain: a review. *Frontiers in Aging Neuroscience*.

[35] Zhan X., Stamova B., Jin L. W., DeCarli C., Phinney B., Sharp F. R. (2016). Gram-negative bacterial molecules associate with Alzheimer disease pathology. *Neurology*.

[36] Hewel C., Kaiser J., Wierczeiko A. (2019). Common miRNA patterns of Alzheimer’s disease and Parkinson’s disease and their putative impact on commensal gut microbiota. *Frontiers in Neuroscience*.

[37] Fasano A., Not T., Wang W. (2000). Zonulin, a newly discovered modulator of intestinal permeability, and its expression in coeliac disease. *Lancet*.

[38] Sapone A., de Magistris L., Pietzak M. (2006). Zonulin upregulation is associated with increased gut permeability in subjects with type 1 diabetes and their relatives. *Diabetes*.

[39] Choi S. H., Aid S., Kim H. W., Jackson S. H., Bosetti F. (2012). Inhibition of NADPH oxidase promotes alternative and anti-inflammatory microglial activation during neuroinflammation. *Journal of Neurochemistry*.

